# Yixin-Fumai granules modulate autophagy through the PI3K/AKT/FOXO pathway and lead to amelioration of aging mice with sick sinus syndrome

**DOI:** 10.1186/s12979-024-00439-y

**Published:** 2024-07-06

**Authors:** Lianzi Jin, Ping Hou

**Affiliations:** 1 Liaoning University of Traditional Chinese Medicine, Shenyang, 110000 China; 2 Department of Cardiology, Affiliated Hospital of Liaoning University of Traditional Chinese Medicine, Shenyang, 110000 China

**Keywords:** Yixin-Fumai granules, Sick sinus syndrome, Autophagy, Aging, PI3K/AKT/FOXO path

## Abstract

**Objective:**

By employing network pharmacology alongside molecular docking techniques, we can delve into the intricate workings of Yixin-Fumai granules (YXFMs) and their impact on sick sinus syndrome (SSS) within wrinkles mice. Specifically, we aim to understand how YXFMs enhance autophagy through the PI3K/AKT/FOXO path.

**Methods:**

The active ingredients and medicinal uses of Ginseng, *ligusticum wallichii*, Ophiopogon, Schisandra, salvia, and astragalus were compiled using the BATMAN-TCM database. We also used Genecards, OMIM, and Disgenet files to identify the disease goals. A hierarchical diagram of “disease-drug-key targets” was generated using the Cytoscape programs. In addition, we established a target protein interaction (PPI) network using the STRING database. Then, the Cluster Profiler R package was used to conduct GO functional enrichment evaluation and KEGG pathway enrichment analyses of the targets. Based on the PPI system, we chose the top communicating targets and substances over molecular docking. In vivo studies were performed to validate these selections further. The mouse model was induced to study the damaged sinoatrial node (SAN) in mice with lower heart rates due to age-related changes. Electrocardiogram and Masson staining assessments were performed to obtain the results. The transmission electron microscope was used to assess the autophagy level of SAN cells. Western blot was employed to analyze the impact of YXFMs on protein expression in the PI3K/AKT/FOXO signaling process throughout SSS therapy in aging mice.

**Results:**

One hundred forty-two active ingredients, 1858 targets, 1226 disease targets, and 266 intersection targets were obtained. The key targets of the PPI network encompassed TP53, AKT1, CTNNB1, INS, and TNF, among others. According to GO functional analysis, the mechanism underlying YXFMs in SSS treatment may primarily be associated with the control of ion transport across membranes, cardiac contraction, regulation of blood circulation, and other biological processes. Based on the results of KEGG pathway enrichment analysis, it was determined that they were mainly enriched in multiple pathways of signaling such as the PI3K-Akt signaling route, MAPK signaling process, AGE-RAGE signaling path, FOXO signaling path, HIF-1 signaling process, and several other paths. Molecular docking demonstrated that five compounds had excellent binding to the key candidate target proteins AKT1 and INS. Through the in vivo studies, we noticed notable effects when administering YXFMs. These effects included the suppression of aging-induced SSS, a decrease in the R-R interval, a rise in heart rate, a reduction in fibrosis, a boost in the autophagy process level, and a spike in the levels of expression of key protein molecules in the PI3K/AKT/FOXO signaling path.

**Conclusion:**

This research has made preliminary predictions about the potential of YXFMs in treating SSS. It suggests that YXFMs may have the ability to target key proteins and critical paths associated with the condition. Further testing has been conducted to discover new findings and evidence of ideas for tackling SSS triggered by aging.

## Background

The human population is entering an era characterized by an aging era. Furthermore, the morbidity and mortality of Sick Sinus Syndrome (SSS) are increasing with aging [1]. Research has shown a correlation between the prevalence of SSS and advancing age [2]. SSS is a clinical syndrome of slow heart rate due to SAN dysfunction pacing or sinus conduction. A decreased heart rate can result in insufficient blood supply to the brain [3, 4], heart, kidney, and other organs, which can cause corresponding symptoms, including palpitations, fatigue, dizziness, and other symptoms. In extreme circumstances, syncope can lead to sudden death. Currently, the application rate of cardiac pacing therapy for its treatment is extremely low due to technical, economic, and human factors, only 10/100,000 person-times in China, whereas the developed countries in Europe and America have reached 100/100,000 person-times.

Most existing biotherapy techniques concentrate on biological pacing technology, which primarily relies on ion channel overexpression; the effect remains unsatisfactory, and there are severe side effects. Western medicine is not suitable for long-term oral administration in the treatment of sick sinus syndrome due to its high side effects.

Traditional Chinese medicine (TCM) has a long history of utilizing herbal formulations to address various health concerns, including cardiovascular diseases [5]. Additionally, TCM has formed a flawless theoretical framework for SSS treatment. However, traditional Chinese medicine has high requirements for doctors’ syndrome differentiation technology, and there are numerous disadvantages to clinical application, namely, the difficulty of preserving decoction and the poor compliance of patients. Consequently, searching for a Chinese patent medicine with precise clinical efficacy and simple administration has become a significant breakthrough in the therapy for SSS.

In the realm of ancestral Chinese medicine, sick sinus syndrome has no corresponding disease name, which typically belongs to the categories of palpitation, shortness of breath, chest stupor, vertigo, fainting [6], etc. From a biological perspective, the primary signs include palpitation, shortness of breath, dizziness [7], and occasionally collapse. Pulse syndrome is characterized by cognitive impairment and is classified as delayed, sluggish, or irregular heart rate [8]. According to the principles of traditional Chinese Medicine, the heart is of utmost importance in governing the functions of the five zang-fu organs. It also oversees the operation of the primary blood vessels, while the pulse is a vital indicator of the heart’s condition.

Consequently, the heart’s deficiency must be reflected in the pulse condition [6]. According to the Theory of Impotence in Plain Questions, when the body experiences Yang deficiency due to aging or weakness from prolonged illness, there is a corresponding deficiency in Yang qi and Yin blood. This leads to weakness in heart qi, resulting in symptoms such as dizziness, fatigue, palpitations, chest tightness, chest pain, amaurosis, syncope, and other manifestations of blood stagnation and inadequate nourishment to the body.

YXFMs is a traditional Chinese medicine compound granule for SSS. The prescription is made of Ginseng, *ligusticum wallichii*, ophiopogon, Schisandra, salvia, and astragalus [9], and it has gained excellent clinical outcomes.

However, given the intricate structure of Chinese patent medication, our present understanding of the drug’s mechanism effectiveness remains uncertain. Network pharmacology revolves around the relationship between illness, genes, and drugs of interest [10]. In addition, network pharmacology is a novel study technique that combines computer science with systems biology. Network pharmacology can be a powerful tool for exploring and elucidating complex TCM formula’s mechanisms of action. Network pharmacology revealed an association between Buyang Huanwu Decoction (BYHWD), a traditional Chinese medicine, in reducing inflammatory factors like IL-6, IL-1β, and MMP9 expression in Myocardial fibrosis [5]. Another study highlights the effect of combining Astragalus Membranaceus and Angelica sinensis as Chinese herbal medicines in treating atherosclerosis through an anti-inflammatory approach by utilizing network pharmacology to identify and validate the underlying molecular mechanisms [11]. Also, network pharmacology contributes to understanding how traditional Chinese medicine can be utilized in modern oncology for treating cervical and lung cancer [12, 13]. Another study identified 77 common targets of luteolin as an active compound in many Chinese herbal medicines, influencing microglia polarization, and mapped a core protein network of 38 proteins. Gene Ontology (GO) and Kyoto Encyclopedia of Genes and Genomes (KEGG) analyses highlighted pathways related to inflammatory response and interleukin (IL)-17 and tumor necrosis factor (TNF) signaling [14].

The present research explores the pharmacodynamic material foundation and potential process of YXFMs by applying molecular docking in network pharmacology. Additionally, it seeks to evaluate the regulating impact of YXFMS on SSS in elderly mice by employing models of animals to offer insights into the clinical management of SSS.

## Pharmacology in networks and molecular docking

### Yixin-Fumai granules’ active component and target screening

The active components and targets were screened with “ginseng, *ligusticum wallichii*, Ophiopogon, Schisandra, salvia and astragalus” as keywords through BATMAN - TCM [15] (http://bionet.ncpsb.org.cn/batman-tcm/index.php), in which Score > = 20; The UniProt database (https://www.uniprot.org/) was utilized to obtain a specific set of YXFMs genes following gene symbol annotation [16].

### Screen the disease targets

Genecards database [17] (https://www.genecards.org), OMIM database [18] (https://www.omim.org), DisGeNet database [19] (https://www.disgenet.org) were used to search Sick Sinus Syndrome–related genes. The criteria for screening data in the GeneCards database was a Relevance score ≥ 11.93(mean value). Besides, the OMIM database did not perform data screening, and the criteria for screening data in the Disgenet database was Score_gda ≥ 0.09(average). In order to achieve SSS targets, the illness goals from all three databases were combined, and any duplicate information was removed.

### Construct the “drug-component-target” network

Use the Venny analysis platform (https://bioinfogp.cnb.csic.es/tools/venny/) to analyze the active ingredient targets of YXFMs and the disease targets of SSS. This will help to identify the common targets between them. Subsequently, the typical targets were employed as screening conditions to determine the primary active components of YXFMs. Cytoscape3.7.2 [20] A software tool created the network model that connects drugs, active ingredients, and targets. Nodes symbolize individual drugs, components, and targets, while edges denote their connections.

### Construct the PPI network

Retrieve the intersection targets from the String database [21] (https://string-db.org), focusing on the species of interest, “Homo sapiens,” and selecting a value of moderate confidence (0.400) [14]. In addition, the PPI structure of YXFMs performing on SSS was acquired and brought into Cytoscape for graph optimization. R software generated a bar graph displaying the top 30 core genes.

### Gene enrichment analysis

Utilizing R software and the underlying database, “org.Hs.eg.db,” to retrieve gene IDs for possible goals. Then, the “DOSE,” “clusterProfiler,” and the “pathview” packages from Bioconductor will be employed to perform GO functional enrichment analysis on those targets. The evaluation includes Biological Process enrichment (BP), Cellular Component enrichment (CC), Molecular Function enrichment (MF), and KEGG functional enrichment analysis [10].

### Molecular docking

The active ingredient’s structural calculations were obtained via the Pubchem database and formatted employing AutoDockTools-1.5.6 software. Protein receptors were obtained from the PDB database, a valuable resource for researchers in this field. The amounts of water and phosphates in the amino acids were eliminated with the assistance of PyMOL programs. In addition, the protein’s active pocket was determined using AutoDockTools-1.5.6 software. Ultimately, the Vina script was employed to dock molecules.

## Experimental verifications

### Animals

A total of 18 C57BL/6 mice (male or female, > 18 months of age, accompanied by a substantial decrease in heart rate of > 20%) utilized to simulate SSS were split into three distinct categories, with six mice in each group. Furthermore, six mature mice (3 months old, male or female) with usual heart rates were chosen as controls. The mice were sourced from Liaoning Changsheng Biotechnology Company (China, Production License SCXK2020-0001) and raised according to the guidelines established by the Experimental Animal Center of China Medical University for a week.

There were two sections to the animal research; one was to look at autophagy in SSS mice and see how YXFMs helped them, and the other was to see if the fundamental process held.

The initial step was to assign mice to one of three groups: control, SSS, or YXFMs. Electrocardiograms were used to record the R-R interval and heart rate after 28 days of gavage with different substances. Mice in the YXFMs group were given YXFMs at a dosage of 1 g/kg/d for 28 days, whereas mice in the other groups were given filtered water at the same dose. Mice were slaughtered using the cervical dislocation procedure, and then the tissues of their hearts were taken.

The second stage included assigning the mice to the following categories: CON, SSS, YXFMs, or SC79. The SC79 group of mice were administered 1 g/kg/d YXFMs intraperitoneal gavage for 28 days, whereas the control group got 40 mg/kg/d SC79 via intraperitoneal injection. After 28 days, the mice were killed using the cervical dislocation technique, and the tissues of their hearts were collected.

The China Medical University Animal Care and Use Committee approved using all mice.

### Masson

The heart tissue was sectioned into paraffin blocks for analysis. The paraffin slices underwent dewaxing and were subsequently rinsed with water that had been distilled. Following the dewaxing and scrubbing process, the nuclei were stained using Regaud’s hematoxylin staining solution over a duration of 10 min. Afterward, they were carefully washed with water and then given another rinse with water that had been distilled to complete the process. Finally, the nuclei were stained using Masson’s compound staining solution for another 10 min. Immerse the thin slices in a buffer of 2% (v/v) glacial acetic acid for a period, followed by a 5-minute immersion in a 1% dodecamolybdenum phosphate solution to achieve distinction. Afterward, a brief stain using aniline blue was applied over 5 min. Next, immerse it in a solution of 0.2% glacial acetic acid over some time. Ultimately, each section underwent dehydration using 95% ethanol, followed by absolute ethanol, and ultimately attached with xylene, an opaque and unbiased gum. Observation of the level of fibrosis in the SAN of mice in every group was conducted using a light microscope.

### Transmission electron microscope

The samples of SAN were fixed using a solution of 3% glutaraldehyde and 1.5% paraformaldehyde over 24 h. After being rinsed with a solution of phosphate buffer of 0.1 M (pH = 7.4), fixation was accomplished by adding 1% osmic acid at 4 °C for two hours. Subsequently, another wash via 0.1 M phosphorous buffer (pH = 7.4) was performed. The specimens underwent dehydration using a series of ethanol solutions with increasing concentrations, saturated in graded acetone, and then placed on an ultramicrotome to create 60–80 nm-thick ultrathin sections. Finally, the slices were treated with uranyl acetate and lead citrate. An electron microscope was utilized to gather and examine the images.

### WB

The use of Western blotting allowed for the detection of Atg8, Atg12, Beclin-1, p62, LC3-II, and INS, INSR, AKT, p-AKT, PI3K, p-PI3K, FOXO, Ace-FOXO, and SIRT1 in mouse SAN. Before homogenizing and lysing in RIPA buffer, the SAN connectives were washed with PBS. The samples underwent centrifugation at 12,000 rpm for 10 min, maintaining a temperature of 4 degrees Celsius. The protein was extracted and quantified using the BCA method provided by Servicebio in China. Following extraction and quantification, the protein content underwent separation using 10% sodium dodecyl sulfate-polyacrylamide gel electrophoresis (SDS-PAGE). Next, a PVDF membrane was used to carry the proteins. The membrane was then incubated at ambient humidity for one hour with a 5% skim milk solution to block it. Primary antibodies targeting different proteins were incubated with the membranes overnight at four °C, after which they were rinsed with TBST. The following genes were sourced from Servicebio, China: Atg8, Atg12, Beclin-1, p62, LC3-II, INS, INSR, AKT, p-AKT, PI3K, p-PI3K, FOXO, Ace-FOXO, SIRT1, and β-tubulin. After four washes with TBST, the cell membranes were incubated at ambient room temperature for an hour with HRP-conjugated AffiniPure goat anti-rabbit IgG from Servicebio, China. Finally, the results will be observed using the ECL chemiluminescence kit Servicebio of China provides.

### Statistics

Three times, each experiment was conducted. Information was evaluated analytically using SPSS (version 19.0) and presented as mean ± standard deviation (mean ± SD). Using one-way analysis of variance (ANOVA) for statistical analyses, differences were considered statistically significant when *P* values were less than 0.05.

## Results

### Potential targets of Yixin-Fumai granules in the treatment of SSS

BAT-TCM platforms screened a hundred and forty-two different YXFMs that might be active, and 1858 targets were obtained after predicting and eliminating duplicates.

Based on the search keyword “Sick Sinus Syndrome”, the data were searched in Genecards, OMIM, and Disgenet databases, and 1226 targets of cerebral ischemia were obtained following retrieving and removing duplicates.

**Fig. 1:**
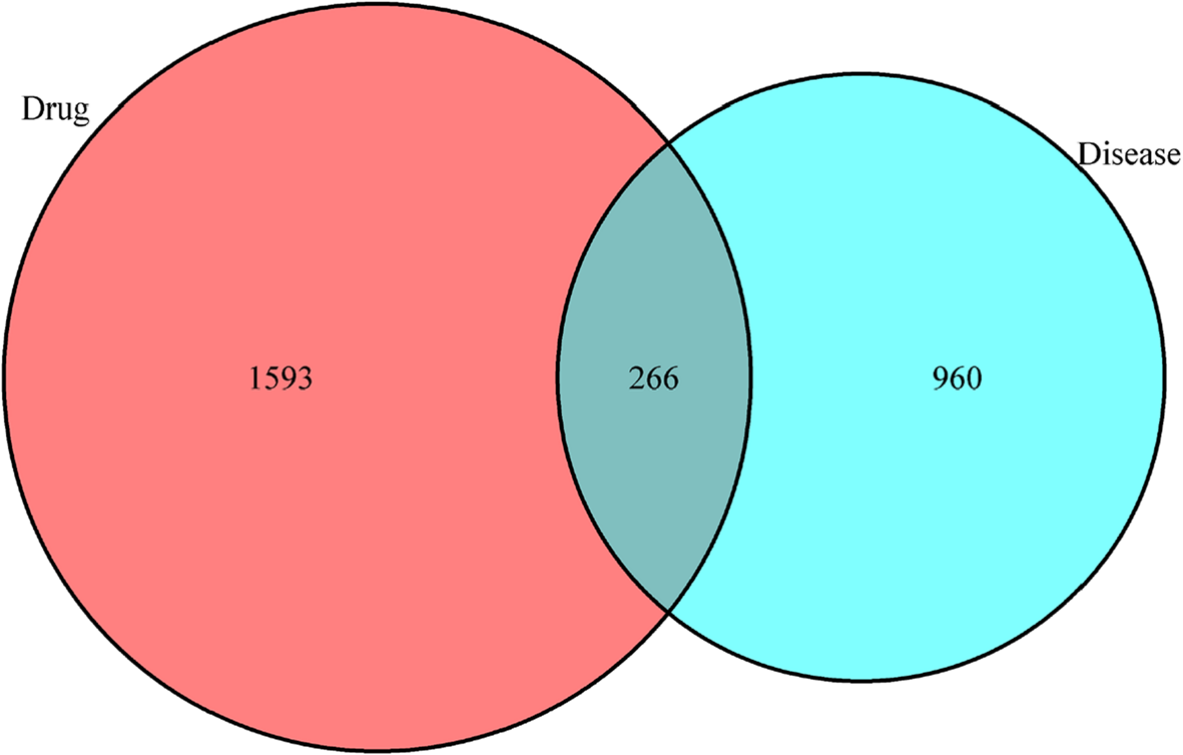
The junction points between YXFMs and SSS vector representation

**Fig. 2:**
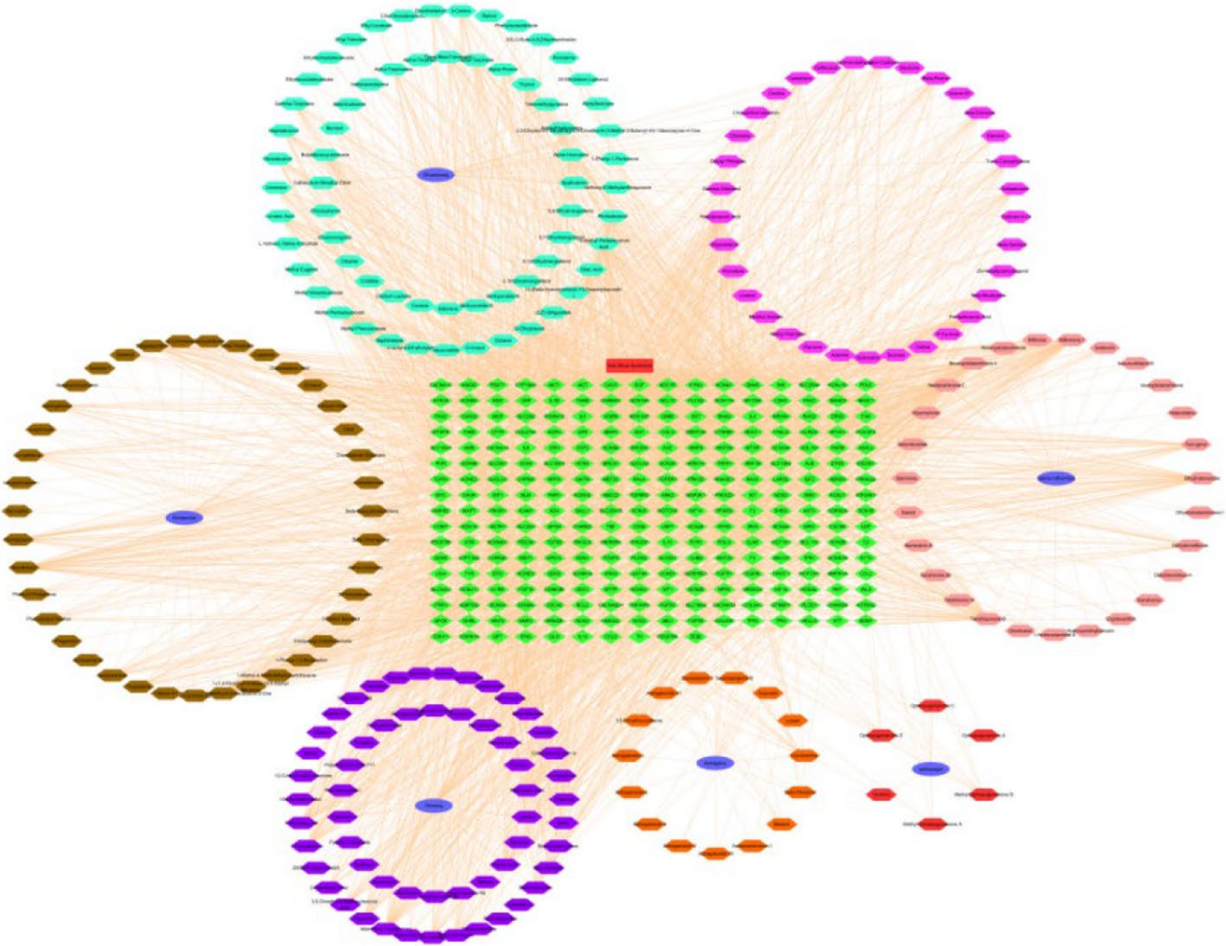
“Disease-drug-component-target” network diagram

The active ingredient targets of YXFMs and disease targets were introduced into the Venny analysis platform to obtain the intersection targets. There are 1,858 gene medications, 1,266 genetic disorders, and 266 genes that interact with both types of pharmaceuticals (Fig. 1). In addition, the primary compounds in YXFMs are identified, and a hierarchical model called “drug-active ingredients-target” was created using the Cytoscape3.7.2 program. This model consists of 262 nodes and 4276 edges (Fig. 2). The findings reveal that the initial eight substances identified in the study are Miltionone I, Neocryptotanshinone II, Tanshiquinone B, Dihydrokaranone, Nootkatone, (E)-9-Isopropyl-6-Methyl-5, 9-dodecene-2-1, and 13-Methyl Pentadecanoic Acid.

**Fig. 3:**
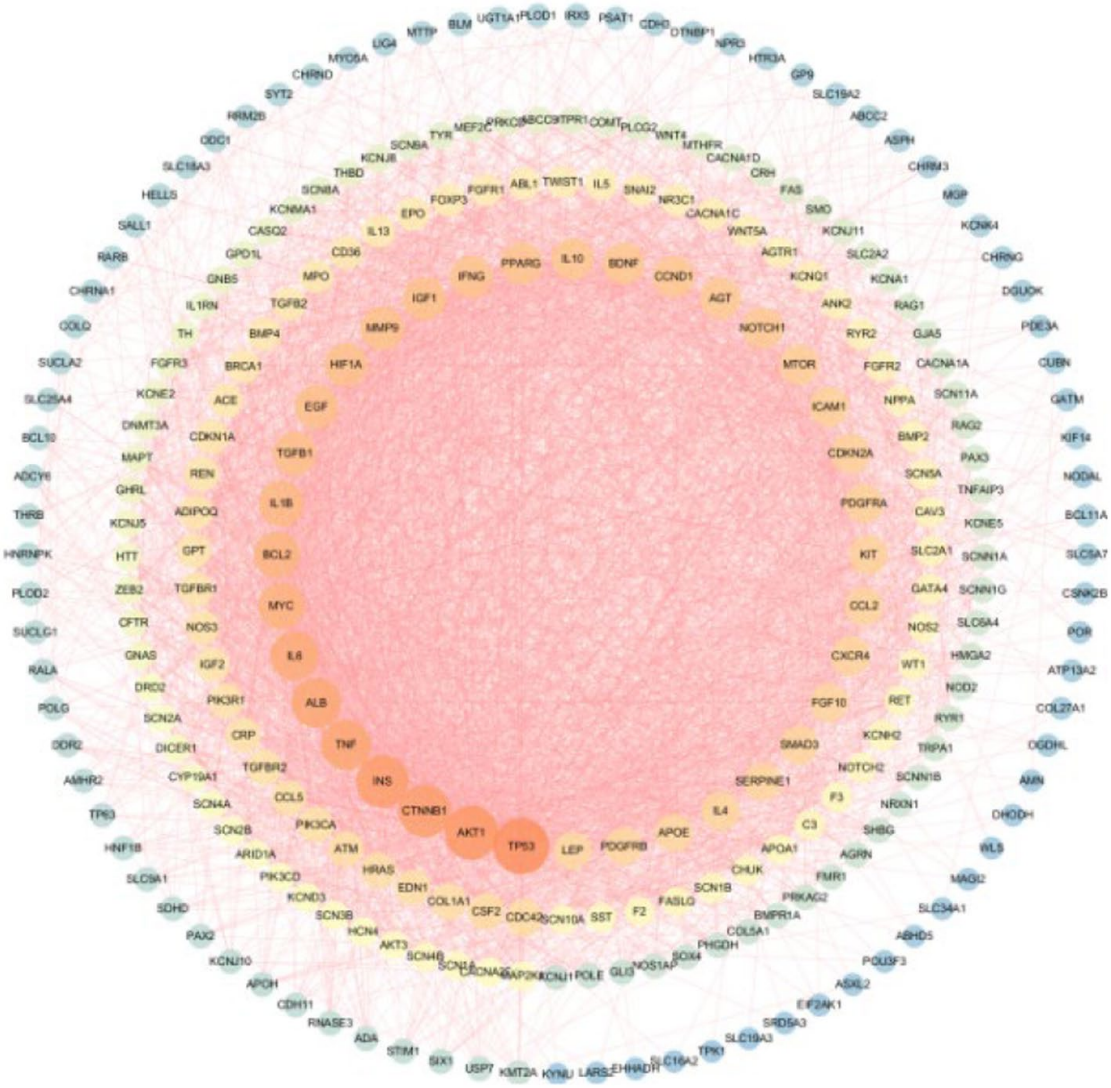
YXFMs’ PPI network for SSS treatment

**Fig. 4:**
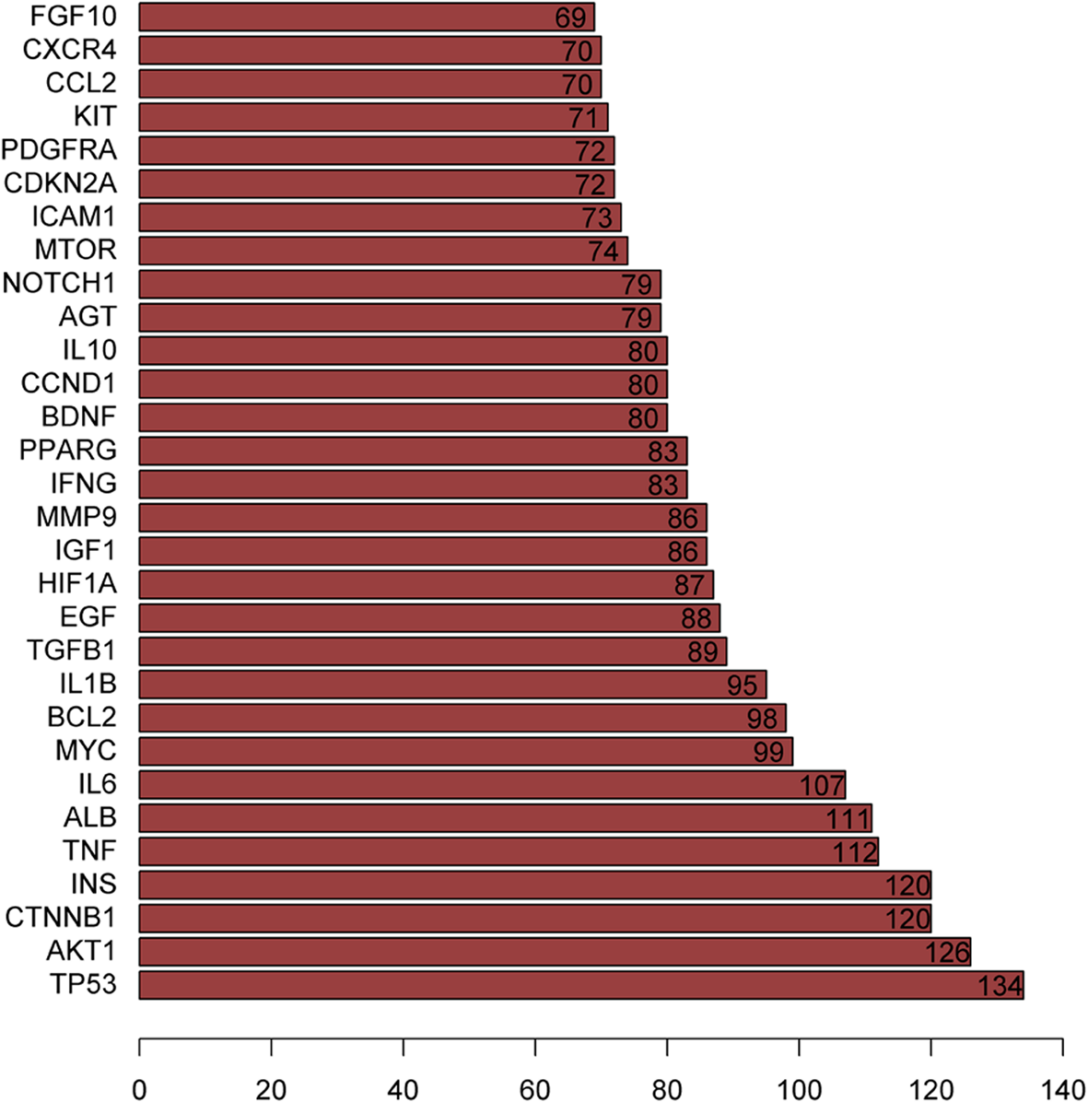
The key targets of YXFMs

The target protein interaction network was obtained by querying the STRING database with the common target data of medications and illnesses. An interaction score of at least 0.4 was required. The PPI data was loaded into Cytoscape 3.7.2 to build a PPI network [9] (Fig. 3). The level value, which indicates the significance of a node in a network, increases as the graph becomes brighter and more prominent. Using R software, PPI data were imported to know the number of connected nodes of core genes, and the bar chart of the top 30 core genes were drawn, which included TP53, AKT1, CTNNB1, INS, TNF, ALB, IL6, MYC, BCL2, and IL1B etc. (Fig. 4).

### Bioinformatics analysis based on target genes

**Fig. 5:**
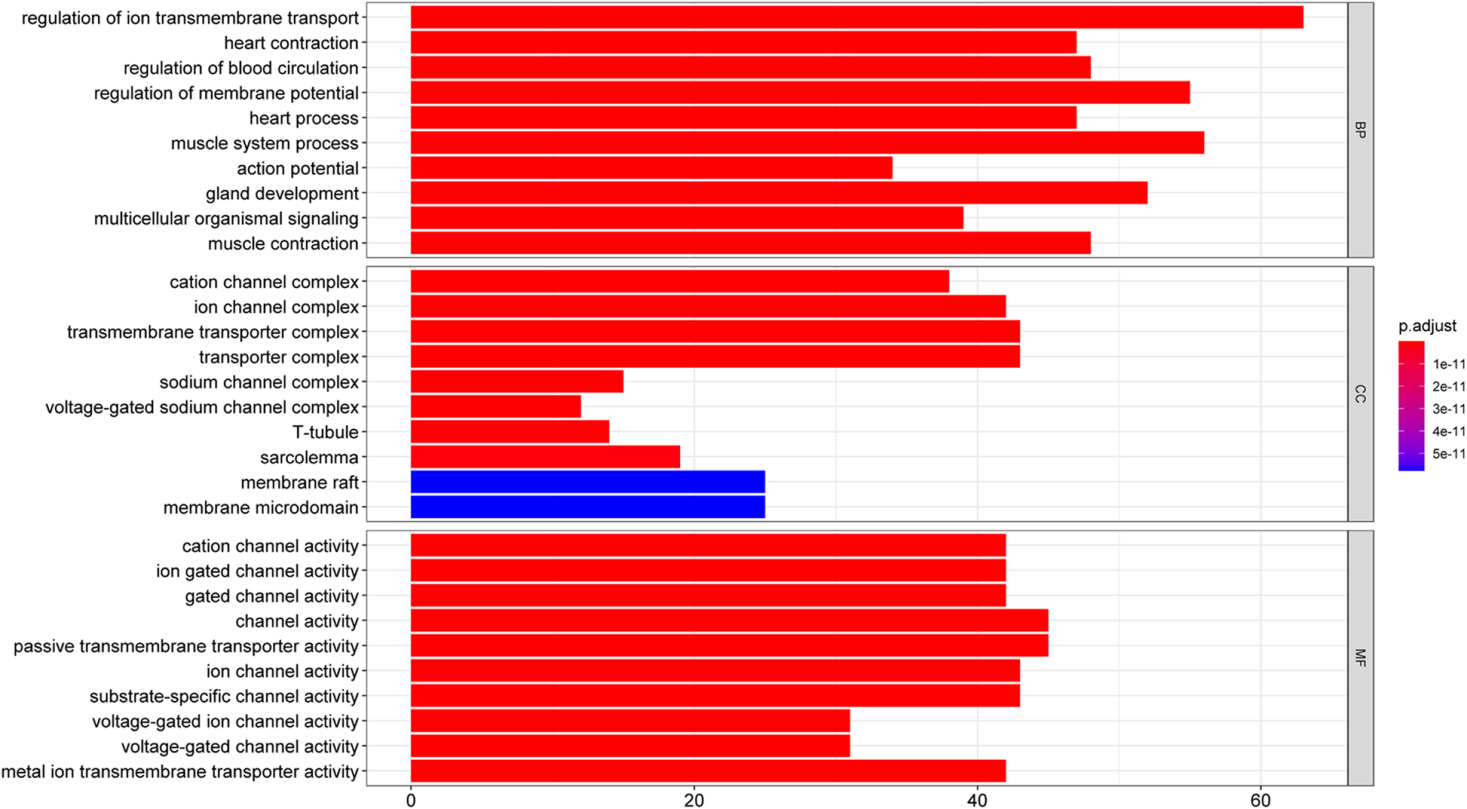
GO enrichment analysis of YXFMs

To identify possible targets, we used the R program plus the underlying database “org. Hs.eg.db” to catalog genes. Subsequently, a Bioconductor performed GO functional enrichment analysis on these potential targets. Each category was ranked according to significance, and bar graphs represented the top 10 enrichment entries (Fig. 5). The analysis revealed that YXFMs played a role in regulating ion transmembrane transport, heart shrinkage, blood flow, cation channel complex, ion channel complex, channel activity, ion-gated channel action, and more. The line length suggests the amount of the genes enriched in GO, and the color reflects the importance of enrichment.

**Fig. 6:**
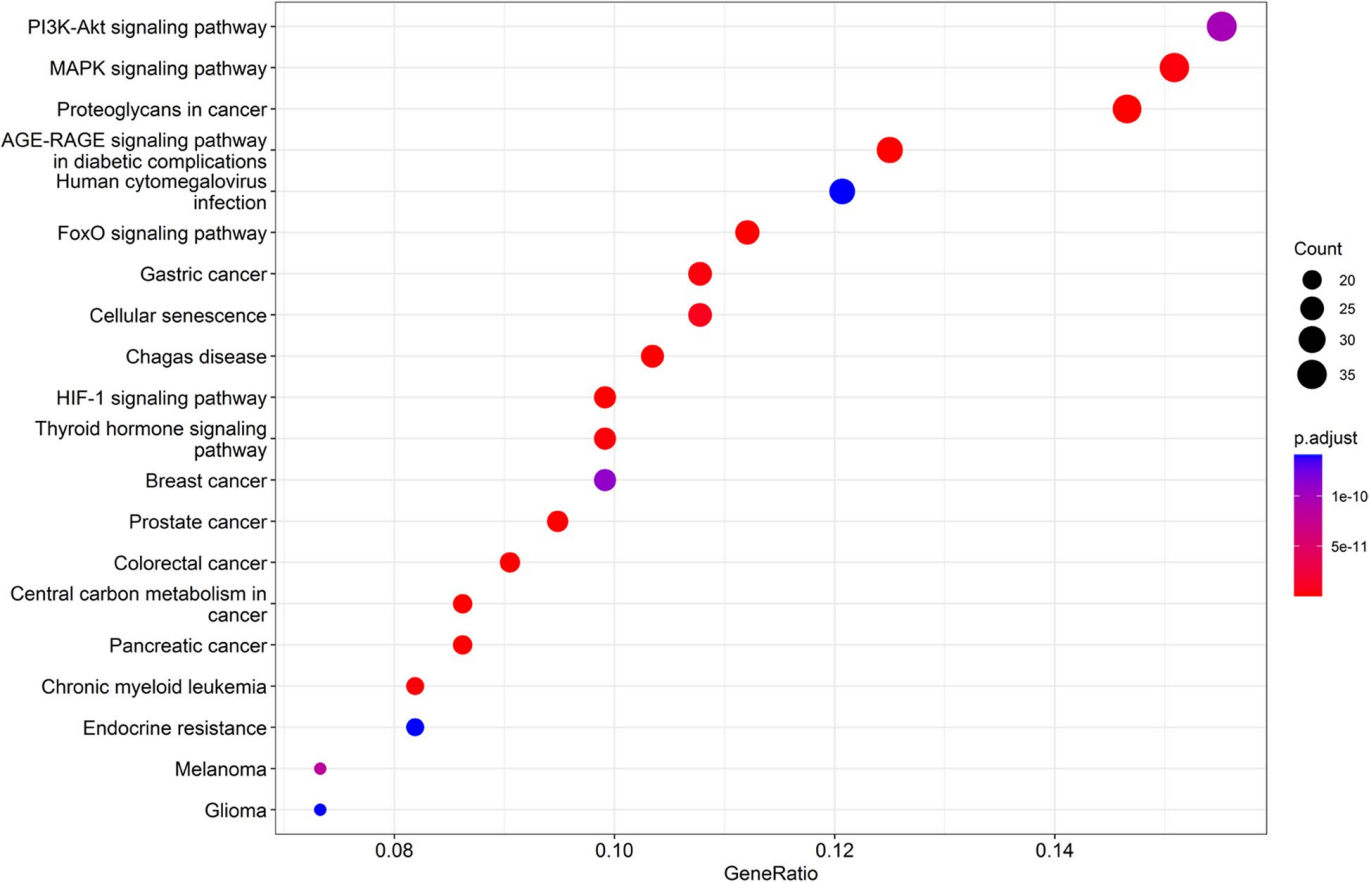
KEGG pathway enrichment analysis of YXFMs

**Fig. 7:**
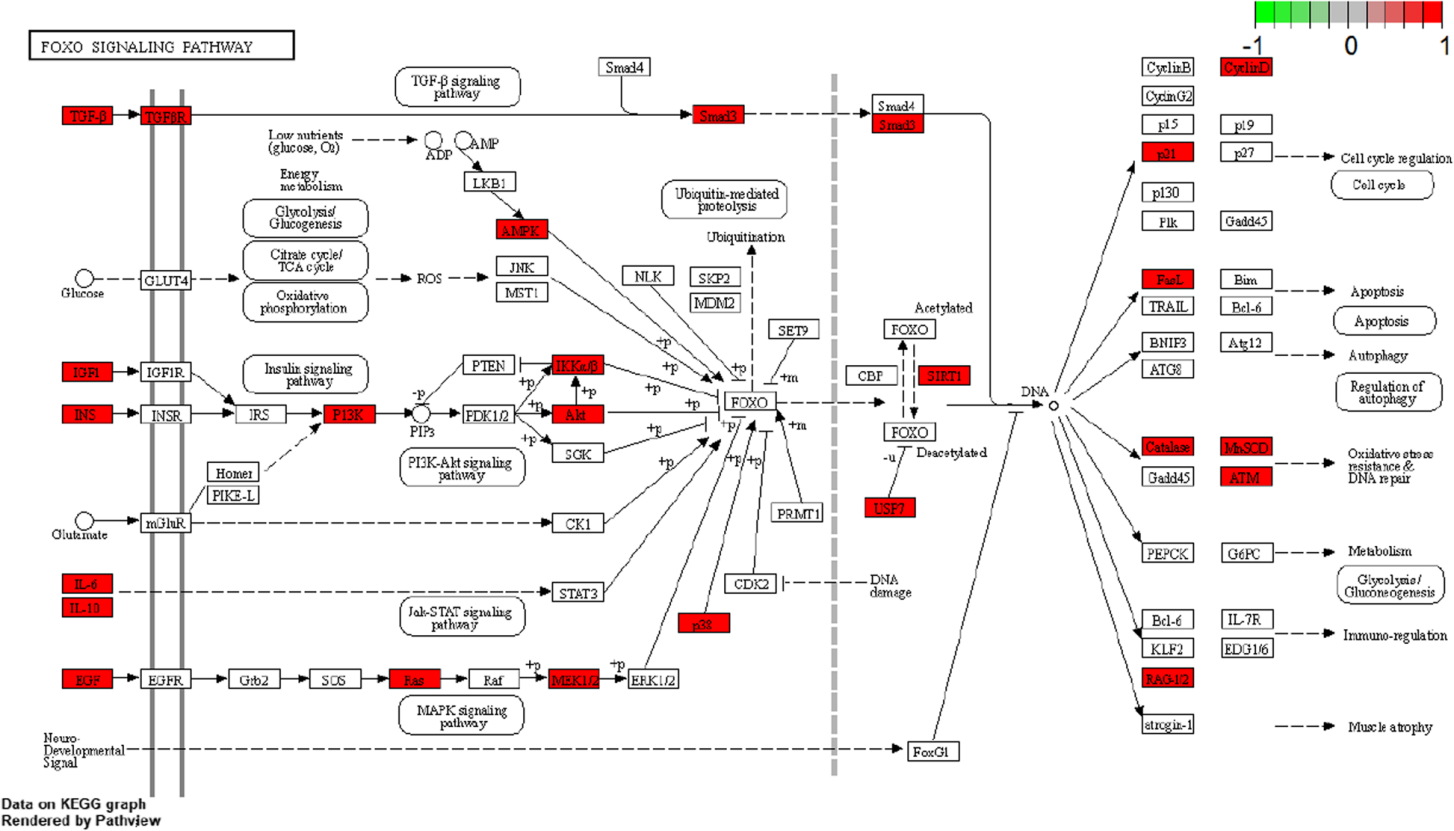
FOXO pathway map is the most significantly enriched pathway

The gene ID of desired targets was obtained using R software and the associated database “org.Hs.eg.db”. Besides, Bioconductor was utilized to perform KEGG function enrichment analysis of these potential targets. According to their significance, 20 enrichment items were rated in the top spot, and a bubble plot was created to display them (Fig. 6). The findings show that YXFMs primarily target specific signaling pathways in treating SSS, including the PI3K-Akt, MAPK, AGE-RAGE, FOXO, HIF-1, and Thyroid hormone signaling pathways. The active parts of YXFMs could target many signaling pathways in their effort to cure SSS. One essential mechanism that regulates the whole network is the PI3K/Akt/FOXO signaling path; this suggests that YXFMs may influence the SSS development process via this pathway. On the autophagy-enriched pathway, we further screened to obtain the autophagy mechanism of the INS-PI3K-Akt-FOXO-SIRT1 signaling pathway (Fig. 7).

### Molecular docking

**Fig. 8:**
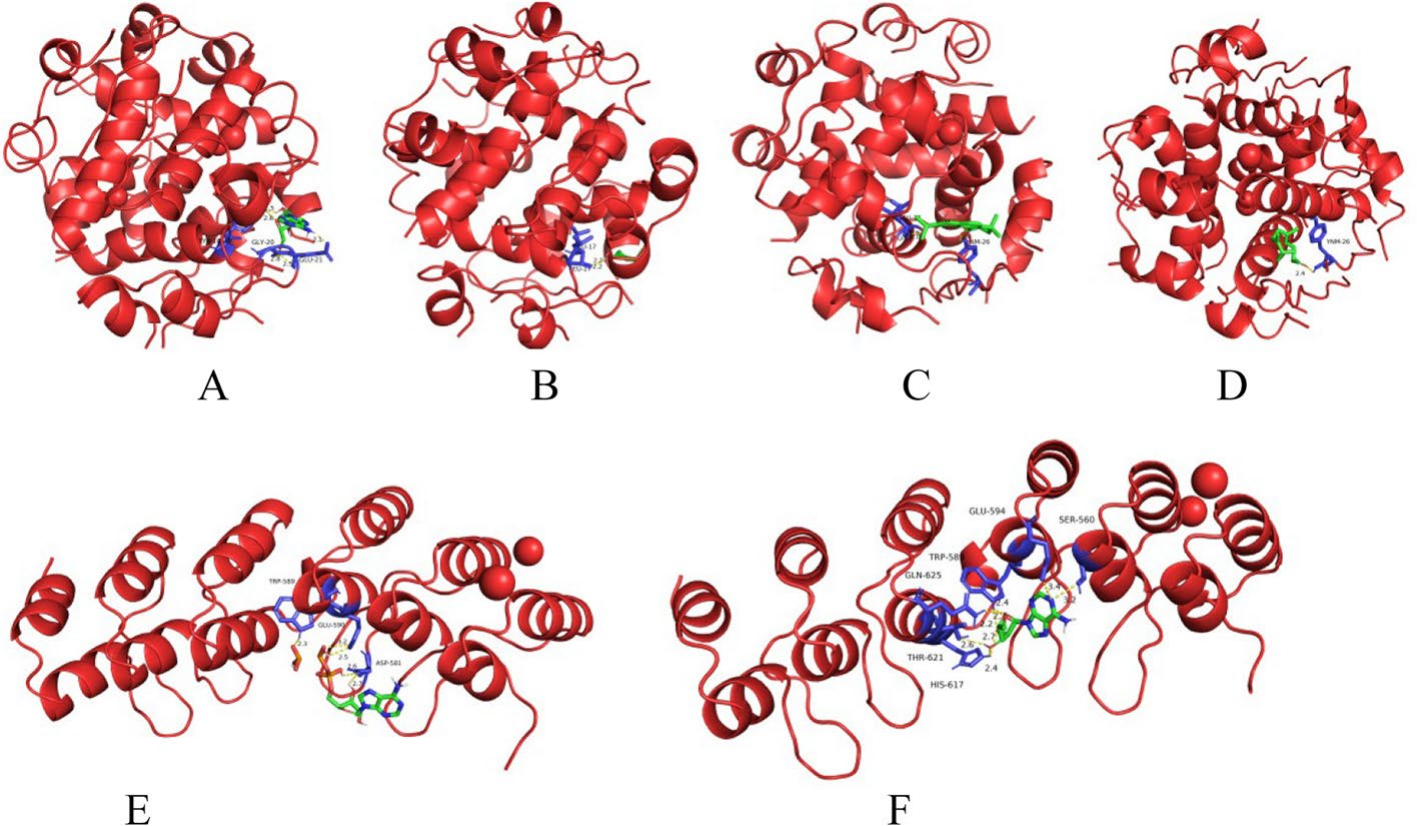
Complex-INS/AKT1 molecular docking shown in three dimensions

 Combining the component-target network relationship and core target screening results, a variety of components in YXFMs, including Adenosine, Neocryptotanshinone Ii, Miltionone I, (E) -9-isopl-6-methyl-5, 9-decadiene-2-one, and Adenosine Triphosphates were found to be strongly associated with the core targets AKT1 and INS, and A total of five compounds were chosen for molecular docking experiments using their corresponding targets. Besides, the minimum binding energy ≤-5.0 kJ·mol-1 shows excellent docking impact of the drug molecule and the protein. The findings showed that these chemicals could dock with AKT1 and INS with stable energies (Table 1). The 3D molecular docking findings were generated using the PyMOL program [12] (Fig. 8).

**Table 1:**
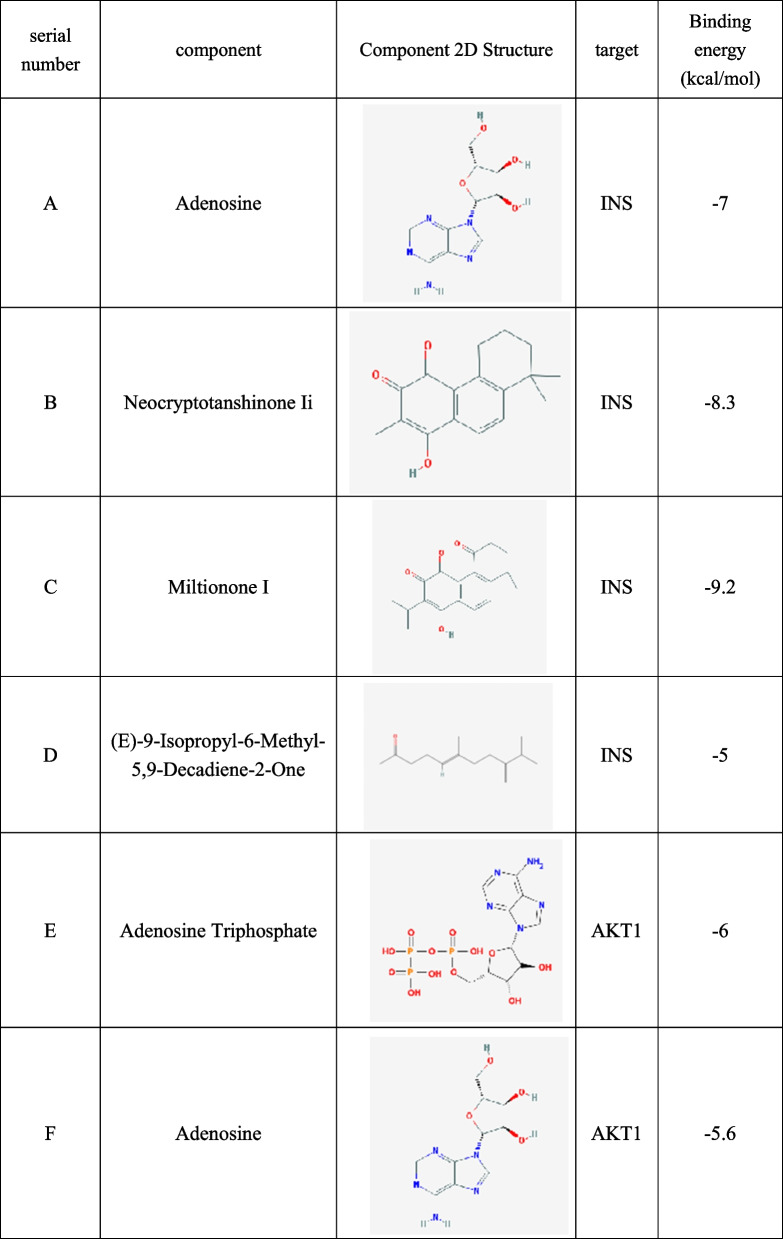
The chemical formulas and results of molecular docking

### YXFMs improved SAN function and increased heart rate in SSS mice

**Fig. 9:**
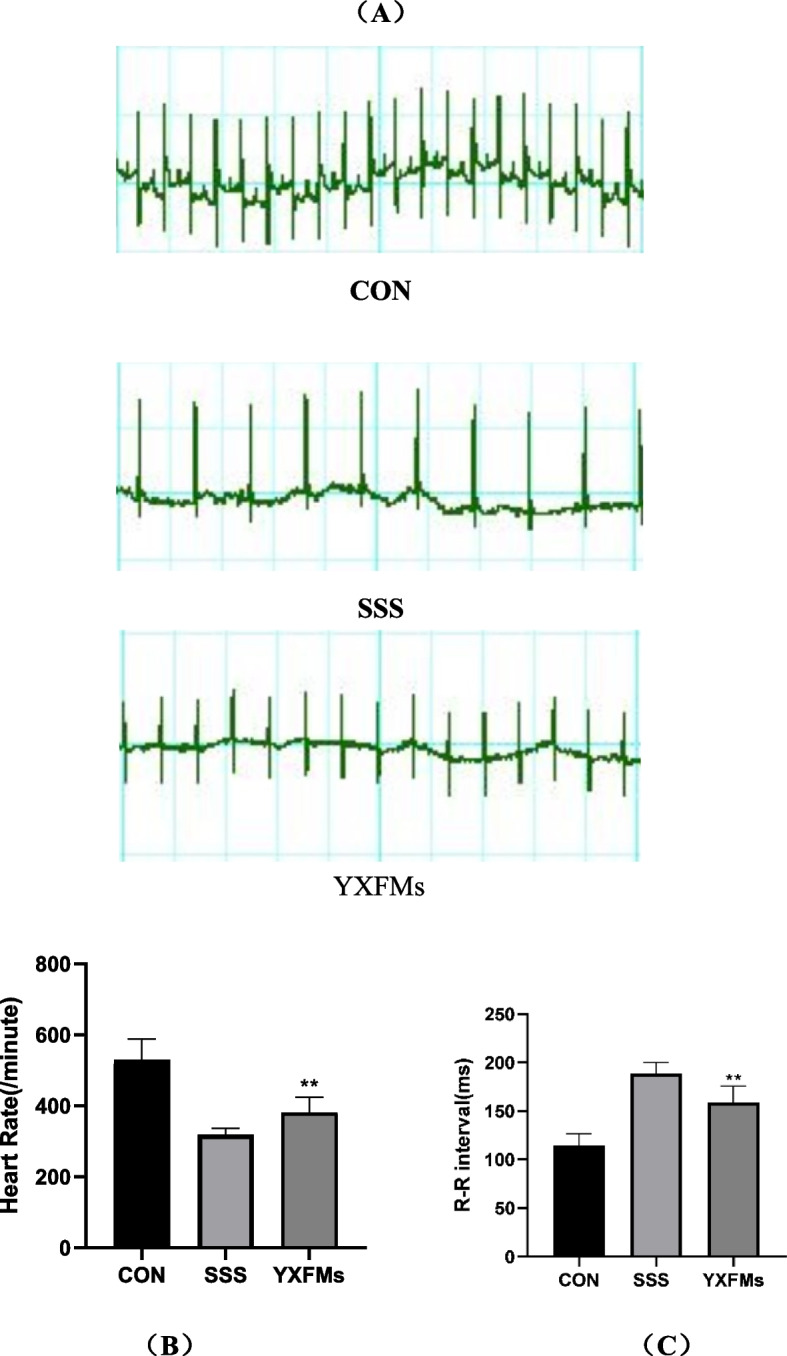
YXFMs enhanced SAN function and led to an increase in heart rate in SSS mice. **A** Heart rates at rest for each mouse group (125 ms/div) CON, SSS, YXFMs groups, respectively from up to down. **B** A statistical evaluation of the heart rates of the mice in each group. **C** Statistical evaluation of the R-R intervals of the ECG in every mouse group. **Compared to SSS, **P < 0.01. The control group is negative. SSS stands for sick sinus syndrome model group. Yixin-Fumai granule therapy group (YXFMs)

**Fig. 10:**
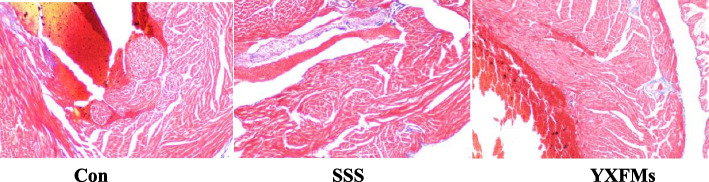
The SAN region of CON, SSS, and YXFMs groups was stained with Masson’s trichrome

 To begin the animal investigation, the SAN function in mice was assessed using Masson staining and an electrocardiogram (resting heart rate and R-R interval). The ECG showed that YXFMs mice had a considerable improvement in heart rate compared to (Control group) CON mice, but the heart rate of SSS animals fell considerably with age (Fig. 9). The Masson staining showed that the CON group had much less SAN fibrosis than the SSS group and that the YXFMs group had even more SAN fibrosis than the SSS group (Fig. 10). Finally, YXFMs significantly decreased age-related SAN fibrosis while simultaneously improving SAN function.

### YXFMs increased autophagy in SAN of SSS mice

**Fig. 11:**
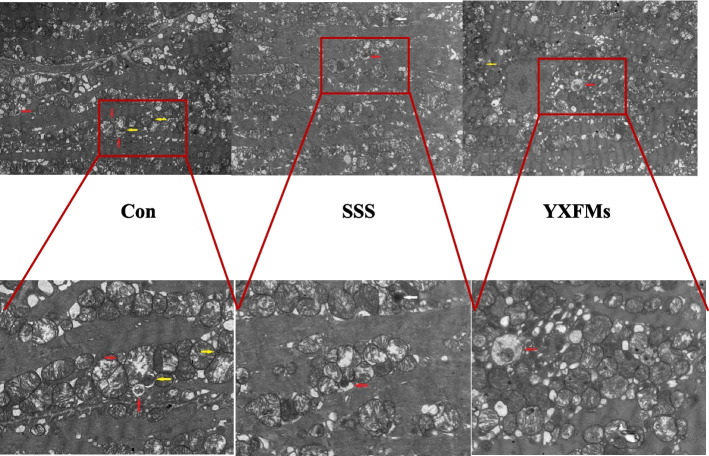
The SAN region of CON, SSS, and YXFMs groups was observed via electron microscopy. Five micrometers and two micrometers serve as the scale bars

Transmission electron microscopy showed that as SSS mice aged, the amount of autophagy and the amount of autophagosomes in their SANs were reduced. Nonetheless, the level of autophagy in the YXFMs group was significantly improved, and lysosomes (Ly), autophagolysosomes (ASS), and autophagosomes (AP) were visible (Fig. 11). Finally, in aging-induced SSS, YXFMs considerably enhanced SAN function and raised SAN autophagy levels.

### YXFMs increased the level of autophagy protein in SAN of SSS mice

**Fig. 12:**
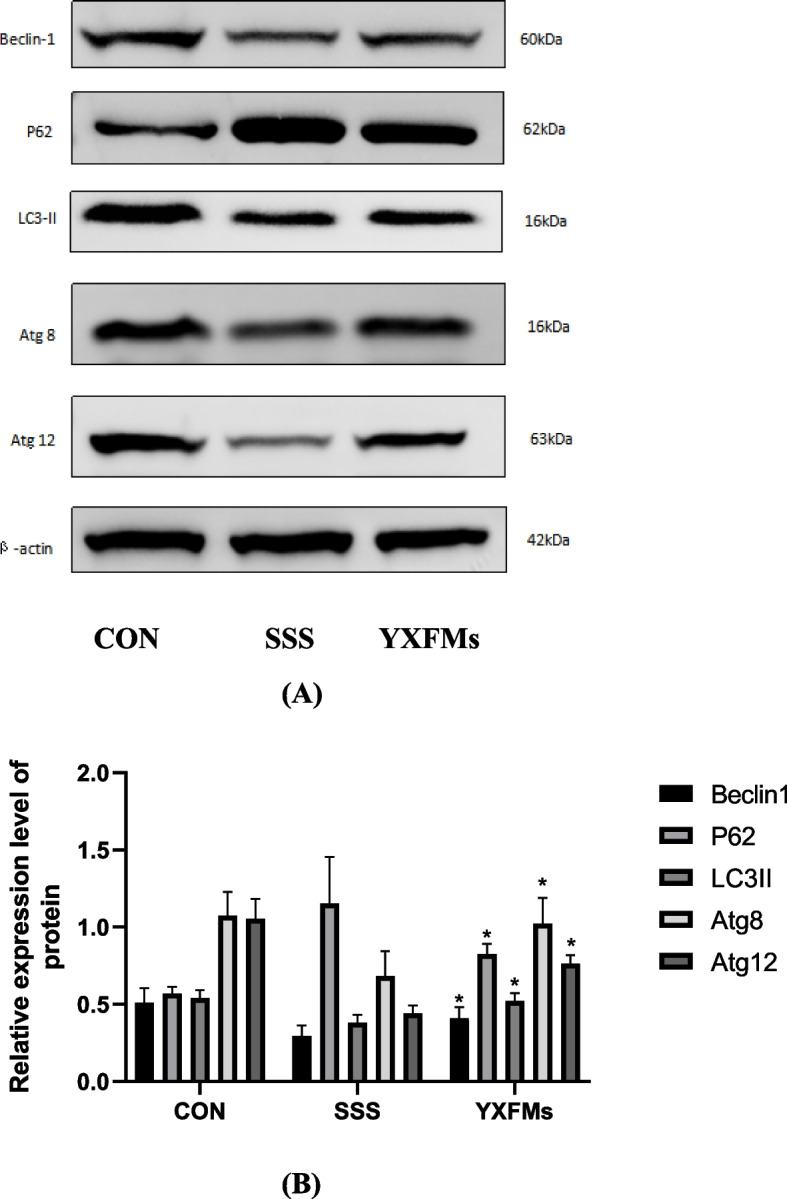
Autophagy in the SAN region of CON, SSS, and YXFMs groups measured by protein assay. **A** The expression of Beclin-1, p62, LC3-II, Atg8, and Atg12 in CON, SSS, and YXFMs groups was measured by Western blotting; **B** The findings of the Western blotting were quantitatively analyzed. *P* < 0.05 in comparison to not using SSS. CON: Control group, The SSS model group represents sick sinus syndrome. YXFMs: Group treated with Yixin-Fumai granules

Autophagy indicator molecules include Beclin-1, with a p62, LC3-II, Atg8, and Atg12. In order to assess the impact of YXFMs upon autophagy, Western blot analysis was used to identify the extent of expression of proteins for Beclin-1, p62, LC3-II, Atg8, and Atg12. A decrease in the protein expression amounts of Beclin-1, LC3-II, Atg8, and Atg12 proteins, as well as an increase in the gene expression level of p62 protein within the SSS category compared to the CON category, suggest the fact that the SSS category impeded the autophagic function of SAN. While p62 protein production was lowered in senile SSS mice treated with YXFMs, Beclin-1, LC3-II, Atg8, and Atg12 protein expression levels were substantially elevated. There were significantly significant differences in protein expression across the groups (Fig. 12), suggesting that YXFMs may increase the autophagic activities of the SANin-aged mice.

### Interactions between YXFMs and the PI3K/AKT/FOXO signals

**Fig. 13:**
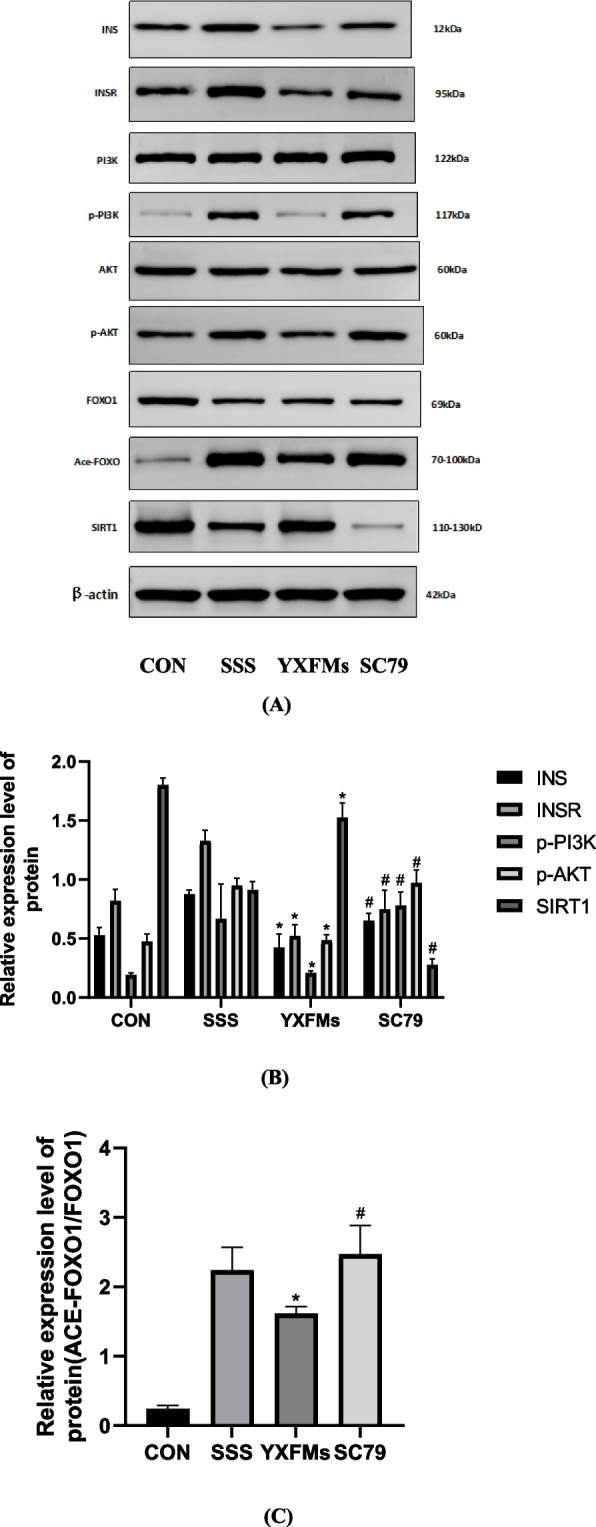
Test of the SAN region PI3K/AKT/FOXO signaling pathway in CON, SSS, YXFMs SC79 groups. **A** The expression of INS, INSR, PI3K, p-PI3K, AKT, p-AKT, FOXO1, Ace-FOXO1, and SIRT1 was detected by Western blotting; **B** Quantitative analysis of the Western blotting findings was performed. #*P* < 0.05 in comparison to SSS. Compared to YXFMs, #*P*<0.05. Downside: the control group. SSS-model group: sick sinus syndrome. YXFMs (Yixin-Fumai granules): group that received therapy.SC79: female mice given YXFMs in addition to SC79 agonist for phosphorylation of AKT (SC79)

In the second part of the animal experiment, the SAN of each group was analyzed using western blotting to determine the expression of INS, INSR, PI3K, p-PI3K, AKT, p-AKT, FOXO1, Ace-FOXO1, and SIRT1. In addition, the amount of INS, INSR, p-AKT/AKT, and p-PI3K/PI3K showed an essential rise in SSS mice. This suggests that the PI3K/AKT route, responsible for activating various cellular processes, became active in SSS mice, leading to the phosphorylation of PI3K/AKT. However, the gene expression levels of INS, INSR, p-AKT, and p-PI3K significantly decreased in SSS mice treated with YXFMs. In the SSS group, there was an important rise in the levels of ACE-FOXO1/FOXO1 and a reduction in the levels of SIRT1 compared to the CON category. In the YXFMs group, a notable enhancement was noticed in the gene expression of Ace-FOXO1/FoxO1 compared to the SSS organization. On the other hand, the AKT phosphorylation agonist SC79 had a counteractive influence on the impact of YXFMs in this context. The differences in the expression of proteins between the categories were statistically significant (Fig. 13).

Thus, YXFMs may increase autophagy efficiency by blocking PI3K/AKT signaling cascade phosphorylation, decreasing FOXO1 acetylation. However, the impact of YXFMs was counteracted through the AKT phosphorylation agonist SC79.

## Discussion

Humanity is currently experiencing a shift towards an aging population, and numerous clinical studies have demonstrated a concerning rise in the prevalence of diseases associated with SAN dysfunction among the elderly (≥ 65 years old) [22]. SSS is primarily attributed to issues with SAN impulse generation as well as the process of conduction. Clinical manifestations of arrhythmias include sinus bradycardia, sinus arrest, sinoatrial block, slow-fast syndrome/tachy-slow syndrome, and Chronotropic dysfunction. Numerous studies have revealed that aging can result in a decline in the maximum heart rate observed in various animal species, such as rats, rabbits, dogs, and cats [23–25]. To develop a scientific understanding of SAN advancing age, we chose mice with a substantially reduced heart rate when they aged and carried out studies mimicking SSS. The findings revealed that the average resting heart rate of the SSS grouping was significantly reduced compared to the CON organization. Additionally, there was an elongation of the R-R interval and a substantial rise in SAN fibrosis in the SSS category, suggesting the effective development of the model. Our research revealed that the YXFMs treatment had a notable impact on the heart rate of SSS mice, effectively reducing SAN fibrosis. The findings suggest that YXFMs can potentially enhance SAN function by reducing fibrosis within SSS mice.

The present investigation employed various scientific techniques, including network pharmacology, molecular docking, and laboratory experimental confirmation, to uncover the intricate workings of YXFMs on SSS. 1858 targets of YXFMs were selected from the database, and the core genes were found through the PPI network to play an essential role in TP53, AKT1, CTNNB1, INS, TNF, ALB, IL6, MYC, BCL2, and IL1B. Molecular docking results show that INS and AKT1 bind well with various compounds. According to KEGG signaling pathway analysis, the PI3K/AKT/FOXO signaling pathway exhibits a significant enrichment. There are many signaling pathways upstream of FOXO that will affect it, making FOXO phosphate Phosphorylated FOXO will be ubiquitinated and degraded, and the undegraded FOXO will enter the nucleus and regulate its transcriptional activity and the expression of downstream genes, thus controlling a range of biological procedures, such as the control of cell cycles, autophagy and apoptosis, oxidative damage, repair of DNA, immune system control, and more [26].

Previous research has indicated that the regulation of FOXO involves various signaling pathways, with the PI3/AKT signaling pathway being particularly significant. Based on the findings from network pharmacology and molecular docking, it is evident that the core target of AKT1 exhibits a solid binding capacity. In typical situations, the FOXO pathway is activated and phosphorylated by The PI3K/AKT pathway, which inhibits its nuclear localization and prevents its dissociation through the target gene promoter region. Consequently, it is incapable of participating in transcriptional control. During periods of stress [27], the PI3K/AKT pathway becomes inactive, causing FOXO transcription factors to relocate from the cytoplasm to the nucleus. Once there, they bind to specific regions on DNA, known as promoter regions. This binding enables them to play a crucial role in the regulation and expression of genes downstream [28]. PI3K/AKT is an important signal transduction pathway that regulates cardiac function. It mainly regulates cardiac function through vascular endothelial growth factor mediating angiogenesis [29], inhibiting myocardial cell apoptosis [30], remodeling the ventricle, and promoting cellular energy metabolism. This signal regulates cardiac function. It plays a critical regulatory role in cardiomyocyte survival and programmed death [31].

Currently, four FOXO homologous genes are discovered in mammals, namely FOX01, FOXOla, FOXO3a, FOX04, and FOX06, located on different chromosomes. FOX01 is one of the more intensively studied subtypes [32]. Our study found that YXFMs reduced SIRT1 enzyme activity, weakened the deacetylation effect of SIRT1 on FOXO1, increased the acetylation level of FOXO1, and inhibited the transcriptional activity of FOXO1, thereby regulating autophagy. Autophagy is one of the biological processes regulated by FOXO. Disruption of autophagy is linked to a range of human ailments, such as overweight or obese people, cardiovascular disease, neurological conditions, and malignancies [33]. Current research indicates that FOXO transcription variables play diverse roles in the control of autophagy [34]. Autophagy is an evolutionarily conserved process crucial to cell homeostasis and survival. It digests and decomposes aging and dead organelles in cells through the lysosomal pathway and ingests various cell substances through endocytosis to gain adaptation. A variety of cellular nutrients required during the body’s survival or life development process to maintain the stability of environmental substances within the entire body’s cells and the balance of cell metabolism [35]. The geriatric science hypothesis proposes that aging phenotypes and age-related diseases share a set of cellular mechanisms [36]. In aging cells, the amount of autophagy becomes lower, which can have an impact on the physiological and fundamental balance of the aging heart. A fully functioning autophagy mechanism is crucial for maintaining the overall balance in the heart as it ages.

To investigate the potential correlation between the protective effect of YXFMs on SAN and autophagy, we devised the initial phase of our animal experiments. Electron microscopy results have confirmed that YXFMs can enhance autophagy in the mouse SAN. The significant improvement in autophagy-related factors such as Beclin-1, p62, LC3-II, Atg8, and Atg12 supports this. These findings suggest that YXFMs can potentially enhance the autophagy level of SAN. For a more comprehensive understanding of the potential mechanism of action of YXFMs, we conducted the second part of our animal experiment. In addition to administering YXFMs, we also injected SC79 into mice.

Interestingly, we observed that SC79 counteracted the effects of YXFMs in vivo. Furthermore, our Western blotting results indicated that YXFMs can decrease The expression of specific genes and proteins in the SAN of mice, which has been found to impact the function of the SAN significantly. Specifically, the levels of INS, INSR, p-PI3K, p-AKT, and SIRT1 have been observed to influence FOXO and acetylated FOXO levels. This, in turn, has been shown to promote autophagy and ultimately enhance the overall function of the SAN. These findings provide further evidence that the PI3K/AKT/FOXO pathway plays a crucial role in the effects of YXFMs and could be a promising therapeutic approach for SSS.

## Conclusion

To summarize, the present research utilized advanced network pharmacology and molecular docking technological advances to investigate the mechanisms and pathways of YXFMs in the treatment of SSS. In addition, in vivo experiments were conducted to validate that YXFMs modulate autophagy via the PI3K/AKT/FOXO pathway, leading to improvements in aging. It results in SAN fibrosis in SSS mice and further enhances SAN function. Nevertheless, this study did not investigate alternative pathways, which does have some limitations.

## Data Availability

The original contributions presented in the study are included in the article.

## Abbreviations

Ace-FOXO1: Acetylated Forkhead Box Protein O1
AGE-RAGE: Advanced Glycation End Products-Receptor for Advanced Glycation End Products
AKT: Protein Kinase B
ANOVA: Analysis of Variance
CON: Control group
Atg8, Atg12, Beclin-1, p62, LC3-II: Proteins involved in the process of autophagy
Cytoscape: An open-source software platform for visualizing complex networks and integrating various types of attribute data
ECG: Electrocardiogram
FOXO1: Forkhead Box Protein O1
Genecards: GeneCards Human Gene Database
GO: Gene Ontology
HIF-1: Hypoxia-Inducible Factor 1
INSR: Insulin Receptor
INS: Insulin
KEGG: Kyoto Encyclopedia of Genes and Genomes
MAPK: Mitogen-Activated Protein Kinase
OMIM: Online Mendelian Inheritance in Man
PI3K/AKT/FOXO: Phosphatidylinositol 3-kinase/AKT/Forkhead box protein O
PDB: Protein Data Bank
PPI: Protein-Protein Interaction
PVDF: Polyvinylidene Fluoride
PubChem: A database of chemical molecules and their activities against biological assays
R: A programming language and software environment for statistical computing and graphics
RIPA: Radio-Immunoprecipitation Assay
SAN: Sinoatrial Node
SC79: AKT phosphorylation agonist
SDS-PAGE: Sodium Dodecyl Sulfate Polyacrylamide Gel Electrophoresis
SIRT1: Sirtuin 1
SSS: Sick Sinus Syndrome
SPSS: Statistical Package for the Social Sciences
STRING: Search Tool for the Retrieval of Interacting Genes/Proteins
TCM: Traditional Chinese Medicine
TP53: Tumor Protein P53
YXFMs: Yixin-Fumai granules

## References

[1] Liu J (2014). Age-associated abnormalities of intrinsic automaticity of sinoatrial nodal cells are linked to deficient cAMP-PKA-Ca(2+) signaling. Am J Physiol Heart Circ Physiol.

[2] Kistler PM (2004). Electrophysiologic and electroanatomic changes in the human atrium associated with age. J Am Coll Cardiol.

[3] Nemati SH (2023). Neuroprotective effects of niosomes loaded with thymoquinone in the cerebral ischemia model of male Wistar rats. Nanomedicine.

[4] Al-Jammal A, Bigdeli MR, Moghadam FM. pH-sensitive oleuropein-loaded niosome: efficient treatment for metastatic brain tumors in initial steps in-vivo. OpenNano. 2022;8:100095.

[5] Wang T (2022). Based on network pharmacology and in vitro experiments to prove the effective inhibition of myocardial fibrosis by Buyang Huanwu decoction. Bioengineered.

[6] Ghorbani M (2024). Tranexamic acid in total hip arthroplasty: an umbrella review on efficacy and safety. J Orthop.

[7] Rahimian Z (2023). Antiseizure effects of *Peganum harmala* L. and *Lavandula angustifolia*. BioMed Res Int.

[8] Li CJ, Fan G, Zhang BL (2012). Successful treatment of sick sinus syndrome with traditional Chinese medicine. Int J Clin Pharm.

[9] Wang T (2022). The mechanism of action of the combination of Astragalus Membranaceus and Ligusticum chuanxiong in the treatment of ischemic stroke based on network pharmacology and molecular docking. Medicine (Baltimore).

[10] Jiang X (2023). Exploration of Fuzheng Yugan mixture on COVID-19 based on network pharmacology and molecular docking. Medicine (Baltimore).

[11] Wang T (2022). Prediction and validation of potential molecular targets for the combination of Astragalus membranaceus and Angelica sinensis in the treatment of atherosclerosis based on network pharmacology. Medicine (Baltimore).

[12] Wang X (2024). The potential mechanism of Guizhi Fuling Wan effect in the treatment of cervical squamous cell carcinoma: a bioinformatics analysis investigation. Medicine (Baltimore).

[13] Wang T (2023). Identification and integration analysis of a novel prognostic signature associated with cuproptosis-related ferroptosis genes and relevant lncRNA regulatory axis in lung adenocarcinoma. Aging.

[14] Wang T (2023). Exploring the mechanism of luteolin by regulating microglia polarization based on network pharmacology and in vitro experiments. Sci Rep.

[15] Liu Z (2016). BATMAN-TCM: a bioinformatics analysis tool for molecular mechANism of traditional Chinese medicine. Sci Rep.

[16] UniProt Consortium. UniProt: a worldwide hub of protein knowledge. Nucleic Acids Res. 2019;47(D1):D506-d515. 10.1093/nar/gky1049 PMC6323992 30395287

[17] Rebhan M (1997). GeneCards: integrating information about genes, proteins and diseases. Trends Genet.

[18] Amberger JS (2015). OMIM.org: online mendelian inheritance in man (OMIM®), an online catalog of human genes and genetic disorders. Nucleic Acids Res.

[19] Piñero J (2017). DisGeNET: a comprehensive platform integrating information on human disease-associated genes and variants. Nucleic Acids Res.

[20] Demchak B (2018). The Cytoscape automation app article collection. F1000Res.

[21] Szklarczyk D (2019). STRING v11: protein-protein association networks with increased coverage, supporting functional discovery in genome-wide experimental datasets. Nucleic Acids Res.

[22] Maillet M, van Berlo JH, Molkentin JD (2013). Molecular basis of physiological heart growth: fundamental concepts and new players. Nat Rev Mol Cell Biol.

[23] Alings AM, Bouman LN (1993). Electrophysiology of the ageing rabbit and cat sinoatrial node–a comparative study. Eur Heart J.

[24] Di Gennaro M (1987). Age-related differences in isolated rat sinus node function. Basic Res Cardiol.

[25] Yin FC (1979). Age-associated decrease in heart rate response to isoproterenol in dogs. Mech Ageing Dev.

[26] Roy SK, Srivastava RK, Shankar S (2010). Inhibition of PI3K/AKT and MAPK/ERK pathways causes activation of FOXO transcription factor, leading to cell cycle arrest and apoptosis in pancreatic cancer. J Mol Signal.

[27] Moghadam FM (2021). TISS nanobiosensor for salivary cortisol measurement by aptamer Ag nanocluster SAIE supraparticle structure. Sens Actuators B Chem.

[28] Vogt PK, Jiang H, Aoki M (2005). Triple layer control: phosphorylation, acetylation and ubiquitination of FOXO proteins. Cell Cycle.

[29] Moghadam FM, Rahaie M (2019). A signal-on nanobiosensor for VEGF165 detection based on supraparticle copper nanoclusters formed on bivalent aptamer. Biosens Bioelectron.

[30] Wang T (2023). Efficacy of aquatic exercise in chronic musculoskeletal disorders: a systematic review and meta-analysis of randomized controlled trials. J Orthop Surg Res.

[31] Calcinotto A (2019). Cellular senescence: aging, cancer, and Injury. Physiol Rev.

[32] Carlsson P, Mahlapuu M (2002). Forkhead transcription factors: key players in development and metabolism. Dev Biol.

[33] Kriel J, Loos B (2019). The good, the bad and the autophagosome: exploring unanswered questions of autophagy-dependent cell death. Cell Death Differ.

[34] Cheng Z (2019). The FoxO-autophagy axis in health and disease. Trends Endocrinol Metab.

[35] Luo F (2020). Melatonin and autophagy in aging-related neurodegenerative diseases. Int J Mol Sci.

[36] Kennedy BK (2014). Geroscience: linking aging to chronic disease. Cell.

